# Recurrent Horizontal Transfers of *Chapaev* Transposons in Diverse Invertebrate and Vertebrate Animals

**DOI:** 10.1093/gbe/evu112

**Published:** 2014-05-27

**Authors:** Hua-Hao Zhang, Cédric Feschotte, Min-Jin Han, Ze Zhang

**Affiliations:** ^1^School of Life Sciences, Chongqing University, China; ^2^College of Pharmacy and Life Science, Jiujiang University, China; ^3^Department of Human Genetics, University of Utah School of Medicine

**Keywords:** horizontal transfer, *Chapaev*, viruses, parasite–host interaction

## Abstract

Horizontal transfer (HT) of a transposable element (TE) into a new genome is regarded as an important force to drive genome variation and biological innovation. In addition, HT also plays an important role in the persistence of TEs in eukaryotic genomes. Here, we provide the first documented example for the repeated HT of three families of *Chapaev* transposons in a wide range of animal species, including mammals, reptiles, jawed fishes, lampreys, insects, and in an insect bracovirus. Multiple alignments of the *Chapaev* transposons identified in these species revealed extremely high levels of nucleotide sequence identity (79–99%), which are inconsistent with vertical evolution given the deep divergence time separating these host species. Rather, the discontinuous distribution amongst species and lack of purifying selection acting on these transposons strongly suggest that they were independently and horizontally transferred into these species lineages. The detection of *Chapaev* transposons in an insect bracovirus indicated that these viruses might act as a possible vector for the horizontal spread of *Chapaev* transposons. One of the *Chapaev* families was also shared by lampreys and some of their common hosts (such as sturgeon and paddlefish), which suggested that parasite–host interaction might facilitate HTs.

## Introduction

Transposable elements (TEs) are fragments of DNA that can move from one place to a new genomic location in their hosts and often make up a large fraction of eukaryotic genomes (Feschotte and Pritham 2007). TEs are divided into two classes based on transposition mechanisms: Class I or RNA elements transpose via reverse transcription of an RNA intermediate; Class II or DNA elements transpose via a DNA intermediate and most do so using a so-called “cut and paste” mechanism (Craig et al. 2002). Horizontal transfer (HT), known as the exchange of genetic material between isolated species, plays an important role in transposon biology and genome evolution (Schaack et al*.* 2010; Wallau et al. 2012; Ivancevic et al. 2013). The *P* element of *Drosophila* was the first TE shown to have been introduced via HT (Daniels et al. 1990). Virtually all major types of TEs have been shown to be capable of HT in a wide variety of eukaryotes (Bartolomé et al*.* 2009; Schaack et al*.* 2010; Thomas et al. 2010; Wallau et al. 2012; Ivancevic et al. 2013). However, the majority of reported horizontal transposon transfers involves drosophilid flies (Schaack et al*.* 2010). Meanwhile, the extent of this phenomenon remains unclear and the molecular mechanisms underlying HT remain largely mysterious. Two facilitating mechanisms have received support recently: One is host–parasite relationships (Yoshiyama et al. 2001; Gilbert et al. 2010); the other is that DNA viruses can act as transposon vectors (Fleming and Summers 1991; Jehle et al. 1998; Turnbull and Webb 2002; Marquez and Pritham 2010; Schaack et al. 2010; Dupuy et al. 2011; Gilbert et al. 2014).

*Chapaev* transposons represent a relatively new superfamily of DNA transposons, which were first identified in 2007 (Kapitonov and Jurka 2007) and recently affiliated to a larger “megafamily” widespread in eukaryotes dubbed *CMC* for *Chapaev*–*Mirage*–*CACTA* (Yuan and Wessler 2011). Hallmarks of the *Chapaev* transposons are generally 3- to 4-bp target site duplication (TSD), terminal-inverted repeats with the invariable “5-CAC and GTG-3” termini (Kapitonov and Jurka 2007; Yuan and Wessler 2011). As in most DNA transposons, *Chapaev* transposases are characterized by the presence of a conserved “DDE” motif in the predicted catalytic domain as well as additional conserved residues diagnostic of the *CMC* group (Yuan and Wessler 2011). However, the biology and evolution of *Chapaev* transposons remain largely uncharacterized. To expand our knowledge on *Chapaev* transposons, we have carried out a detailed analysis of the characteristics and evolution of three *Chapaev* families, which we identified in a variety of invertebrate and vertebrate species, as well as in an insect bracovirus. We provide evidence that the widespread taxonomic distribution of these elements is the result of multiple HT events likely facilitated by both parasitism and viruses.

## Materials and Methods

### Animal Materials

For the silkworm *Bombyx mori*, strain Dazao was obtained from the State Key Laboratory of Silkworm Genome Biology (China) and its DNA extraction was based on the standard techniques (Nagaraja and Nagaraju 1995). Asian Swallowtail *Papilio xuthus* was purchased from Shanghai Qiuyu Biotechnology Co., Ltd (China). DNA or tissue samples of the Arctic lamprey *Lethenteron camtschaticum*, the Pacific bluefin tuna *Thunnus orientalis*, the channel catfish *Ictalurus punctatus*, the turnip sawfly *Athalia rosae*, and the lizard *Anolis carolinensis* were kindly provided by related researchers (please see Acknowledgments for details). Then, their total DNAs were extracted using TIANamp Genomic DNA Kit (TIANGEN). Meanwhile, quality of DNAs extracted from these species was examined on 1% agarose gel electrophoresis.

### DNA Collection

The assembled *B. mori* genome sequence was downloaded from Silkworm Genome Database (SilkDB version 2, http://www.silkdb.org/silkdb/doc/download.html, last accessed June 10, 2014). The Monarch butterfly *Danaus plexippus* genome resource (version 2) was obtained from MonarchBase (Zhan and Reppert 2013) through the web site at http://monarchbase.umassmed.edu/resource.html (last accessed June 10, 2014). The triatomine bug *Rhodnius prolixus* genomic supercontig sequences were downloaded from VectorBase (Lawson et al*.* 2009) at http://www.vectorbase.org (last accessed June 10, 2014). The whole-genome shotgun (WGS) sequences of the sea lamprey *Petromyzon marinus*, Arctic lamprey, Pacific bluefin tuna, dragonfly *Ladona fulva*, turnip sawfly, tenrec *Echinops telfairi*, and lizard were downloaded from the National Center for Biotechnology Information (NCBI, http://www.ncbi.nlm.nih.gov/, last accessed June 10, 2014).

### Identification and Copy Number Calculation of *Chapaev* Elements

A previously uncharacterized *Chapaev* element (named *Garfield_BM*) was discovered in the silkworm genome when proteins of *Chapaev* elements from Repbase (Jurka et al*.* 2005) were used as queries in tBLASTn (default parameters) (Altschul et al. 1990) searches against the draft genome assembly of the silkworm (Zhang H-H, Zhang Z, unpublished data). The sequences of *Merrow_PM* and *Conan_ET* (see Nomenclature for details) were obtained from Repbase (Kapitonov and Jurka 2007). Then, their nucleotide sequences were used as initial queries (BLASTn [Altschul et al. 1990] using default parameters) to find these *Chapaev* elements in other genomes available at the NCBI, including nucleotide collection (nr/nt), genome survey sequences (GSS), expressed sequence tag (EST), high throughput genomic sequences (HTGS), and the WGS databases (as of September 2013) (Thomas et al. 2010). They were considered in a species if hits were ≥80% identical to the query over at least 300 bp because *Merrow* transposons identified in all teleost fishes were quite short (from 300 to 800 bp) (table 1).

**Table 1:** Characteristics of *Merrow*, *Garfield*, and *Conan* Newly Identified in This Study

Group	Common Names	TE Names	Length (bp)	Copy Number	Exon1 (aa)	Exon2 (aa)	Representatives
Species							
*Merrow*							
*Petromyzon marinus*	Sea lamprey	*Merrow_PM*^a^	2,451	>254	78	484	AEFG01041997
*Lethenteron camtschaticum*	Arctic lamprey	*Merrow_LC*	2,455	>249	78	484	KF965286
*Acipenser transmontanus*	White sturgeon	*Merrow_AT*	807	n/d	—	188	DR976541
*Ictalurus punctatus*	Channel catfish	*Merrow_IP*	833	n/d	—	—	KF965284
*Ictalurus furcatus*	Blue catfish	*Merrow_IF*	661	n/d	—	—	FD224147
*Polyodon spathula*	Mississippi paddlefish	*Merrow_PS*	304	n/d	—	—	JX448770
*Thunnus orientalis*	Pacific bluefin tuna	*MerrowN1_TO*	577	18	—	—	KF965282
*Ladona fulva*	Dragonfly	*Merrow_LF*	1,922	105	75	233	APVN01033993
*Garfield*							
*Bombyx mori*	Silkworm	*Garfield_BM*	2,289	>7	78	478	AADK01000850
		*GarfieldN1_BM*	468	97	—	—	KF965283
*Rhodnius prolixus*	Triatomine bug	*Garfield_RP*	2,289	16	78	478	ACPB02036275
*Cotesia sesamiae Mombasa bracovirus*	Viruses	*Garfield_MB*	2,291	n/d	77	477	EF710639
*Papilio xuthus*	Asian Swallowtail	*Garfield_PX*	1,254	n/d	—	149	KF965285
*Athalia rosae*	Turnip sawfly	*Garfield_AR*	1,328	2	—	306	KF965288
*Danaus plexippus*	Monarch butterfly	*Garfield_DP*	2,092	3	78	478	AGBW01002745
*Conan*							
*Echinops telfairi*	Tenrec	*Conan_ET*^a^	1,865	88	461	—	AAIY02038089
*Anolis carolinensis*	Lizard	*Conan_AC*	3,363	1	—	—	AAWZ02011613
		*ConanN1_AC*^a^	443	40	—	—	KF965287
		*ConanN2_AC*	295	281	—	—	KF965289

Note.—n/d, not determined, as the data were obtained from sequences deposited in the nucleotide collection (nr/nt) database, EST database, GSS database or HTGS database. —, Not found.

^a^*Chapaev* transposons deposited in Repbase, and other *Chapaev* transposons were newly identified in this study.

In order to determine the boundary of these elements, the best hits identified in a species (for which genome sequences were available) were blasted using BLASTn (Altschul et al. 1990) against each genome. Then, these retrieved sequences (identity and coverage >80% of the query sequences) were extracted with 500-bp flanking sequences using our Perl script, and they were aligned using MUSCLE (Edgar 2004) to determine their boundary. In addition, copies (4–50) in each species (supplementary table S1, Supplementary Material online) were also aligned using MUSCLE, and their consensus sequences were reconstructed using the above multiple alignments in each genome using DAMBE (Xia and Xie 2001) after gaps were removed. If one genome sequence contained highly fragmented copies or low copy number (<3), the best hit represented the consensus sequence. Also, if these *Chapaev* elements were identified in a nonsequenced species, the best hit identified in this species was used as the consensus sequence. If these transposons identified in one species were chimaeric, they were excluded from the following analysis.

Next, we used these respective consensus sequences to mask each genome in which *Merrow*, *Garfield*, and *Conan* were identified to estimate copy number. All blast hits with more than 100 bp and 80% identity were used to calculate copy number. Because there are many chimaeric copies in the tenrec genome, only elements that were at least 40% coverage to the consensus sequence were considered in estimating copy number. Three miniature inverted-repeat transposable elements (MITEs) derived from *Merrow*, *Garfield*, and *Conan* were also discovered in Pacific bluefin tuna, silkworm, and lizard. As MITEs are generally <600 bp, size and sequence homogeneity (Feschotte et al*.* 2002), their copy numbers were calculated based on the following criteria: 1) All fragments showed more than 80% identity and coverage to their consensus sequences and 2) fragments were considered to be a single insertion when they were separated by less than 200 bp (Granzotto et al. 2011). Meanwhile, there were two subfamilies of MITEs identified in lizard (*ConanN1_AC* and *ConanN2_AC*) and the length of *ConanN1_AC* was about 150 bp longer than that of *ConanN2_AC*. Therefore, fragments of *ConanN2_AC* were assigned to be a single copy when they were separated by less than 100 bp.

### Sequence Analysis

Potential open reading frame of *Chapaev* elements used in this study was predicted using FGENESH (http://linux1.softberry.com/berry.phtml, last accessed June 10, 2014), GENSCAN (http://genes.mit.edu/GENSCAN.html, last accessed June 10, 2014), or getorf in EMBOSS-6.3.1 package (Rice et al*.* 2000) with the default parameters. Multiple alignments of these elements were created by MUSCLE (Edgar 2004). Shading and minor manual refinements of these aligned sequences were deduced using Genedoc (Nicholas et al. 1997). Each pairwise identity was calculated by Bioedit (Hall 1999) after all ambiguous and gapped sites were removed.

We also downloaded *Chapaev3*-like transposons, which were deposited in Repbase Update (Jurka et al*.* 2005) at Genetic Information Research Institute (http://www.girinst.org, last accessed June 10, 2014). Then, their nucleotide sequences were used as queries to do BLASTN (default parameters) (Altschul et al. 1990) against their respective available whole-genome sequences. All full-length or nearly full-length copies of each element were extracted with 100-bp flanking sequences using our Perl script. These sequences were aligned using MUSCLE to determine their TSD. *Chapaev* elements that only had highly fragment copies in their host genome were not included in this analysis. *Chapaev* paralogous empty sites were identified using the similar method described by previous studies (Marquez and Pritham 2010). *Chapaev* orthologous sites were determined by synteny analysis of 5,000 bp flanking these *Chapaev* transposons insertion sites.

Four data sets (one amino acid transposase sequence data set and three nucleotide sequence data sets) were created and used for phylogenetic analyses. The amino acid database consisted of *Chapaev* elements obtained from Repbase and discovered during the course of this study to determine the phylogenetic diversity of the *Chapaev* transposons. The other three databases of nucleotide sequences were created by respective full-length or nearly full-length copies of *Merrow*, *Garfield*, and *Conan* identified in this study to determine the relationship of copies of the same *Chapaev* element in different hosts.

The amino acid data set was aligned using MUSCLE (Edgar 2004), and a neighbor-joining tree was constructed using MEGA4 (pairwise deletion, Poisson correction model, 1,000 bootstrap replicates; Tamura et al*.* 2007). The remaining three databases were also aligned using the multiple sequence alignment program MUSCLE (Edgar 2004), and all ambiguous sites were manually excluded because there were a few ambiguous sites whereas most transposons from different species were aligned. Then, the best-suited nucleotide substitution models for these data were selected using Akaike information criterion (AIC) in Modeltest3.6 (Posada and Crandall 1998). The best-suited nucleotide substitution models for *Merrow*, *Garfield* and *Conan* were K81uf+G, HKY+G, and TVM+G, respectively. Then, phylogenetic trees were created using MrBayes 3.1.2 software (Ronquist and Huelsenbeck 2003) until the values of the average standard deviation of split frequencies were stably below 0.01.

### Age Analyses and Relative Insertion Periods

The timing of amplification of transposons in each species could be estimated by calculating the sequence divergence between copies and the ancestral sequence and by applying its neutral mutation rate (Waterston et al*.* 2002; Pace and Feschotte 2007). To estimate age of each copies of *Merrow*, *Garfield* and *Conan*, only copies spanning at least 50% of their consensus sequence were used in this analysis (Pagan et al*.* 2010). Then, they were aligned using MUSCLE (Edgar 2004), and the amount of nucleotide substitution (*k*) between each insertion and its respective consensus was estimated using Kimura 2-parameter distance method (Kimura 1980). Then, the insertion time of each element was estimated by the formula *T* = *k*/2*r* (Li 1997), where *T* corresponds to the insertion time in millions of years, *k* corresponds to the number of nucleotide substitutions per site, and *r* corresponds to the neutral mutation rate of the species lineage. If we accept that the elements from each other within a genome evolve neutrally since their insertion, the rate of neutral evolution available for their host nuclear genes might be employed. We used the neutral mutation rates for tenrec (2.9173 × 10^−9^/site/year; Pace et al*.* 2008), sea lamprey (1.9 × 10^−9^/site/year; Kuraku and Kuratani 2006), and Arctic lamprey (1.9 × 10^−9^/site/year; Kuraku and Kuratani 2006). Because a neutral mutation rate is not available for silkworm, we applied an estimated mutation rate previously published in Lepidoptera (1.909 × 10^−8^/site/year; Simonsen et al*.* 2011). Because there is no reliable neutral mutation rate available for other species or their close related taxa, these species were not included in this analysis. The phylogenetic tree of species in this study was based on Timetree of life (Hedges et al. 2006) and Taxonomy in NCBI. Divergence times of species were taken from the literature (Douzery et al. 2004; Peterson et al. 2004; Hedges et al. 2006; Kuraku and Kuratani 2006; Wiegmann et al. 2009). Divergence times between the channel catfish and the blue catfish *Ictalurus furcatus*, between the tobacco hornworm *Manduca sexta* and the silkworm, and between turnip sawfly and the ants are unknown. Therefore, their divergence times depicted in the phylogenetic tree were only for illustrative purposes.

### Testing for Purifying Selection

To test for purifying selection, codon alignments of *Merrow* and *Garfield* as well as elongation factor 1-alpha (*EF-1α*) genes of their hosts were created using PAL2NAL software (http://www.bork.embl.de/pal2nal/, last accessed June 10, 2014; Suyama et al*.* 2006). Because there are no complete coding sequences of transposase of *Conan* transposon in *A. carolinensis* (*Conan_AC*) due to stop codons or frameshifts, *Conan* transposon was not included in this analysis. Then, synonymous (d*s*) and nonsynonymous (d*n*) divergences between them, as well as their ratio (d*n*/d*s*) were calculated using the SNAP tool in the HIV Sequence Database (http://www.hiv.lanl.gov, last accessed June 10, 2014; Korber 2002). Codon bias as determined by the effective number of codon (*N*_c_) value was computed using CodonW (Wright 1990).

In addition, multiple alignments of 40–50 copies (at least 50% coverage to their consensus sequences) *GarfieldN1_BM* (extracted from silkworm), *Conan_ET* (extracted from tenrec), *ConanN1_AC*, and *ConanN2_AC* (extracted from lizard) were used to built neighbor-joining tree in MEGA 4 (Tamura et al*.* 2007), with p-distance model, pairwise deletion and 1,000 bootstrap replicates.

### Polymerase Chain Reaction and Sequencing of *Merrow*, *Garfield*, and *Conan*

To validate the presence of *Merrow*, *Garfield*, and *Conan* identified computationally, their polymerase chain reaction (PCR) primers were designed using their flanking or internal sequences (supplementary table S2, Supplementary Material online). PCR was carried out with an initial denaturation step of 4 min at 95 °C followed by 32–35 cycles of 40 s at 95 °C, 40 s at 55–58 °C, and 2 m at 72 °C. Then, PCR products were run in 1% agarose gels in 1× Tris acetate–ethylenediaminetetraacetic acid buffer and visualized under UV light. Purified PCR products were cloned into PMD-19 cloning vector (TaKaRa). One random clone of each species was selected and sequenced.

### Nomenclature

**Fig. 1.—:**
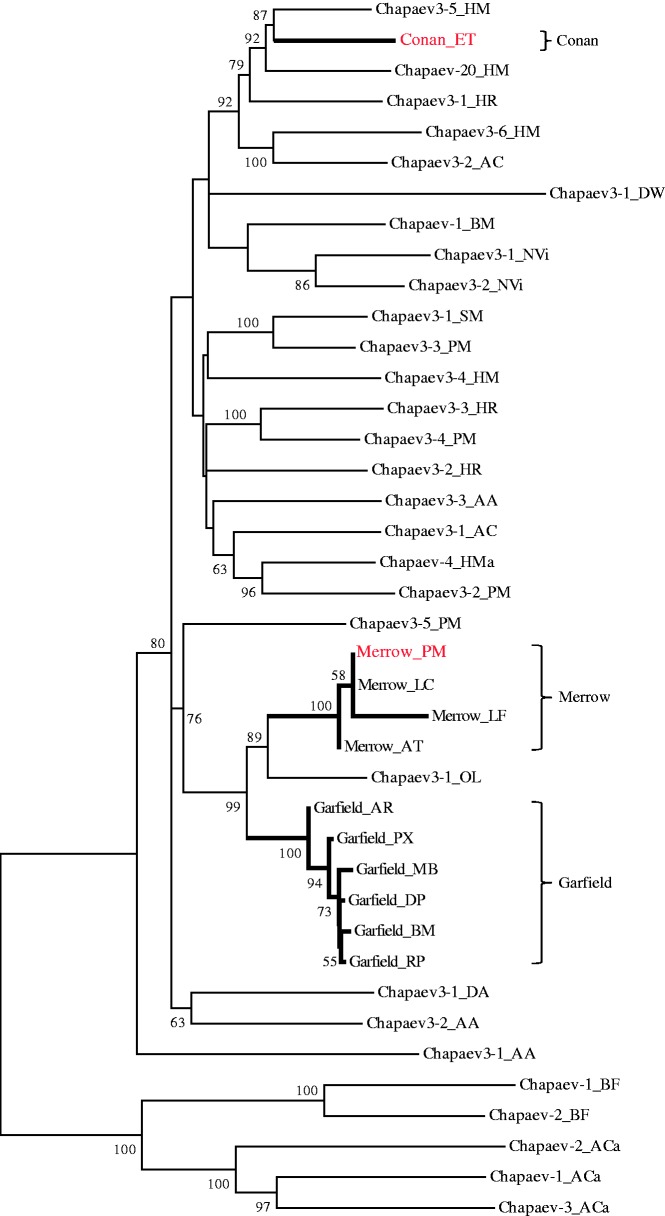
Phylogenetic relationships of *Chapaev3* transposases. The neighbor-joining tree was constructed using MEGA4 (pairwise deletion, Poisson correction model, 1,000 bootstrap replications) based on a multiple alignment of amino sequences of the *Chapaev3* transposases with five representatives (*Chapaev1_BF*, *Chapaev2_BF*, *Chapaev1_ACa*, *Chapaev2_ACa*, and *Chapaev3_ACa*) obtained from Repbase as an outgroup. Bootstrap values >50% were shown. Clusters of *Merrow*, *Garfield*, and *Conan* were displayed using thick line. *Merrow_PM* and *Conan_ET* were deposited in Repbase, and they were shown using red color. However, others were newly identified in this study. Species abbreviations: HM/HMa, *Hydra magnipapillata*; ET, *Echinops telfairi*; HR, *Helobdella robusta*; AC, *Anolis carolinensis*; DW, *Drosophila willistoni*; BM, *Bombyx mori*; NVi, *Nasonia vitripennis*; SM, *Schmidtea mediterranea*; PM, *Petromyzon marinus*; AA, *Aedes aegypti*; LC, *Lethenteron camtschaticum*; LF, *Ladona fulva*; AT, *Acipenser transmontanus*; OL, *Oryzias latipes*; AR, *Athalia rosae*; PX, *Papilio xuthus*; MB, *Cotesia sesamiae Mombasa bracovirus*; DP, *Danaus plexippus*; RP, *Rhodnius prolixus*; DA, *Drosophila ananassae*; BF, *Branchiostoma floridae*; ACa, *Aplysia californica*.

We note that the *Merrow* identified in the sea lamprey *P. marinus* and the *Conan* identified in the tenrec *E. telfairi* and lizard *A. carolinensis* were previously designated with different names in Repbase (Kapitonov and Jurka 2007). However, the Repbase nomenclature for these elements was a potential source of confusion. For example, the sea lamprey *Merrow* family has been named *Chapaev3-1_PM* in Repbase, whereas the *Conan* families described in tenrec and lizard have been named corresponding *Chapaev3-1_ET* and *Chapaev3-3N1_AC*. The Repbase nomenclature would seem to imply that *Chapaev3-1_PM* and *Chapaev3-1_ET* are more closely related to each other than to *Chapaev3-3N1_AC*. However, our results clearly show that *Chapaev3-1_ET* and *Chapaev3-3N1_AC* belong to the same family (*Conan*) whereas *Chapaev3-1_PM* falls within a distinct family (*Merrow*) (fig. 1 and table 1). Furthermore, our result shows that these families were not restricted to these species but are also present in many other species (table 1). Thus for simplicity and clarity, we decided to introduce the corresponding names *Merrow* and *Conan* for these two families. To the best of our knowledge, no members of the *Garfield* family have been characterized previously or deposited in Repbase.

## Results and Discussion

### Identification and Characterization of *Merrow*, *Garfield*, and *Conan*

While investigating DNA transposons in the assembled genome of the silkworm *B. mori*, we discovered a previously uncharacterized family of transposon we designated as *Garfield_BM*. A consensus sequence for *Garfield_BM* was reconstructed by aligning multiple copies extracted from the *B. mori* genome assembly. The consensus length is 2,289 bp long and is predicted to contain two exons encoding a 556 amino acid (aa) transposase (Tpase) (supplementary fig. S1, Supplementary Material online, and table 1). The Tpase displays three highly conserved motifs [C(2)C, LH, and H(4)H] characteristic of the *Chapaev* superfamily of transposons (Yuan and Wessler 2011). Phylogenetic analysis based on a multiple alignment with representatives of the *Chapaev* transposases available in Repbase (fig. 1) places the silkworm transposon within the *Chapaev3* subgroup (Kapitonov and Jurka 2007). In addition, the silkworm *Chapaev* elements were associated with a 3-bp putative TSD of 5′-TWA-3′ consensus sequence (supplementary table S3, Supplementary Material online). We also observed that the nucleotide adjacent to the apparent TSD was always an “A” on the 5′-end and a “T” on the 3′-end (supplementary fig. S2, Supplementary Material online). To determine if these characteristics are shared with other members of the *Chapaev3* group, we also analyzed the insertion bias of *Chapaev3*-like elements deposited in Repbase (Jurka et al*.* 2005) and found that all *Chapaev3* transposons examined were also flanked by “TWA” TSDs and inserted between “A” and “T” (supplementary table S3 and fig. S2, Supplementary Material online). Furthermore, paralogous empty sites (i.e., homologous sites identified within the same genome but lacking the transposon insertion) confirmed that *Chapaev3*-like elements create a “TWA” TSD upon insertion (supplementary fig. S3, Supplementary Material online).

**Fig. 2.—:**
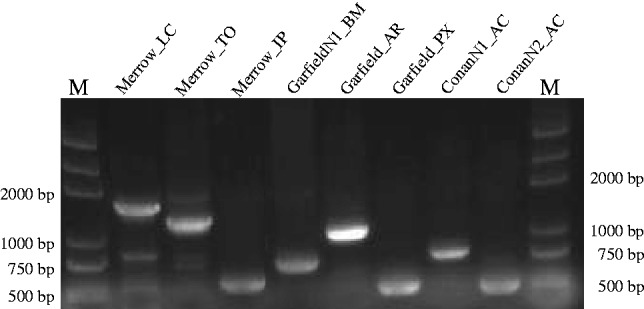
Experimental verification of the presence of *Merrow*, *Garfield*, and *Conan* identified in this study. PCR fragments of the expected sizes were obtained from species studied. All PCR products were confirmed by cloning and sequencing. “M” represents the marker. Species abbreviations: LC, *Lethenteron camtschaticum*; TO, *Thunnus orientalis*; IP, *Ictalurus punctatus*; BM, *Bombyx mori*; AR, *Athalia rosae*; PX, *Papilio xuthus*; AC, *Anolis carolinensis*.

It has been documented that DNA transposons are capable of invading a variety of species by means of HT (Schaack et al. 2010). Because the level of sequence similarity between *Chapaev3* transposases from widely diverged animal species appeared to be inconsistent with the phylogenetic relationships of their hosts (Kapitonov and Jurka 2007), we carried out a detailed investigation of the taxonomic distribution and evolution of *Merrow*, *Garfield*, and *Conan*. We used their consensus sequences as queries in BLASTn (Altschul et al. 1990) searches of all NCBI databases. These searches yielded highly significant hits (e value ranging from 0 to 6 × e^−120^) in a wide range of animal species and in an insect bracovirus (table 1). To rule out database artifacts or contamination, we sought to obtain experimental validation for the presence of these transposons in several of these species by PCR amplification from genomic DNA using primers internal or flanking one of these transposons followed by sequencing of cloned PCR products. We were able to obtain genomic DNA for seven animal species and for all of them confirmed the presence of *Merrow*, *Garfield*, and *Conan* we detected in the corresponding whole-genome assemblies (fig. 2) (GenBank accession numbers KF965282–KF965289).

**Fig. 3.—:**
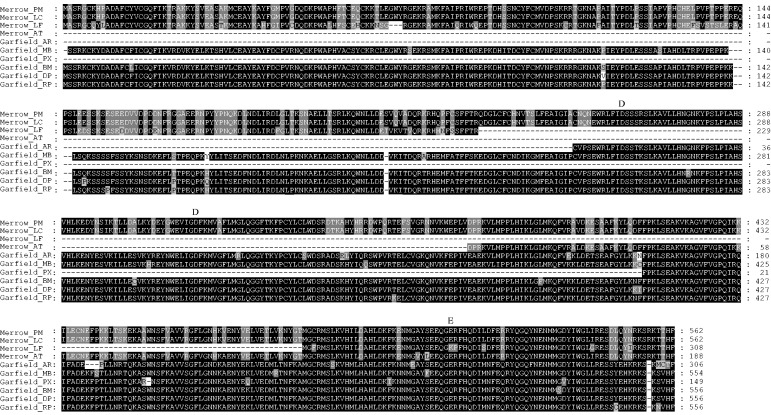
Multiple alignments of consensus sequences of *Merrow* and *Garfield* transposases. An alignment of these elements was created by MUSCLE. Shading and minor manual refinements of these aligned sequences were deduced using Genedoc. The “DDE” catalytic core encoded by their transposases was also shown. Species abbreviations: PM, *Petromyzon marinus*; LC, *Lethenteron camtschaticum*; LF, *Ladona fulva*; AT, *Acipenser transmontanus*; AR, *Athalia rosae*; MB, *Cotesia sesamiae Mombasa bracovirus*; PX, *Papilio xuthus*; BM, *Bombyx mori*; DP, *Danaus plexippus*; RP, *Rhodnius prolixus*.

For each species, we then reconstructed consensus ancestral sequences of *Merrow*, *Garfield*, and *Conan* (see Materials and Methods). Besides consensus sequences of *Merrow_PM* and *Conan_ET*, we also note that another consensus sequence (*ConanN1_AC*) has been deposited previously in Repbase (Kapitonov and Jurka 2007). To the best of our knowledge, all other *Merrow*, *Garfield*, and *Conan* were newly identified in this study (fig. 1 and table 1). Phylogenetic analyses of consensus Tpase sequences confirmed that *Merrow*, *Garfield*, and *Conan* represent three distinct families within the *Chapaev3* group (fig. 1). The phylogenetic analysis also suggested that *Merrow* and *Garfield* were more closely related to each other and might descend from a relatively recent common ancestor (fig. 1). Indeed, both *Merrow* and *Garfield* Tpases are encoded by two exons (fig. 3) whereas most other *Chapaev3* Tpases appear to be encoded by a single exon (Kapitonov and Jurka 2007; data not shown). However, pairwise sequence similarity between any *Merrow* and *Garfield* consensus sequences was still no greater than 66% at the nucleotide level, suggesting that they represent distinct transposon families (Wicker et al. 2007).

### Nonautonomous Elements Derived from *Garfield* and *Conan*

MITEs are a group of nonautonomous elements, which was first discovered in maize (Bureau and Wessler 1992). Generally, MITEs originate from a particular deletion derivative of an autonomous DNA transposon that is subsequently amplified to high copy number to form an homogenous subfamily of nonautonomous elements (Feschotte and Pritham 2007). In this study, we found that two MITE families were direct internal deletion derivatives of one full-length *Chapaev* transposon in the silkworm and lizard (supplementary fig. S4, Supplementary Material online). Our results also showed that *ConanN1_AC* and *ConanN2_AC* were two relatively old subfamilies of lizard nonautonomous transposons as most of their copies were 80–90% identity to their consensus sequences. This is consistent with a previous proposal that members of the *Chapaev* DNA transposon superfamily have long been transpositionally inactive in the anole lizard (Novick et al*.* 2010). By contrast, *GarfieldN1_BM* seems to have experienced a recent burst transposition in the silkworm as all copies shared more than 91% identity to their ancestral sequence (data not shown). This level of divergence would imply a peak of amplification of *GarfieldN1_BM* at about 0.8–1.6 Ma (supplementary fig. S5, Supplementary Material online) based on neutral substitution rates previously estimated for lepidopterans (Simonsen et al*.* 2011). The presence of these MITEs in silkworm and anole lizard was experimentally validated by PCR using their flanking sequences to design primers (fig. 2; GenBank accession numbers KF965283, KF965287, and KF965289).

### Evidence for HTs

**Fig. 4.—:**
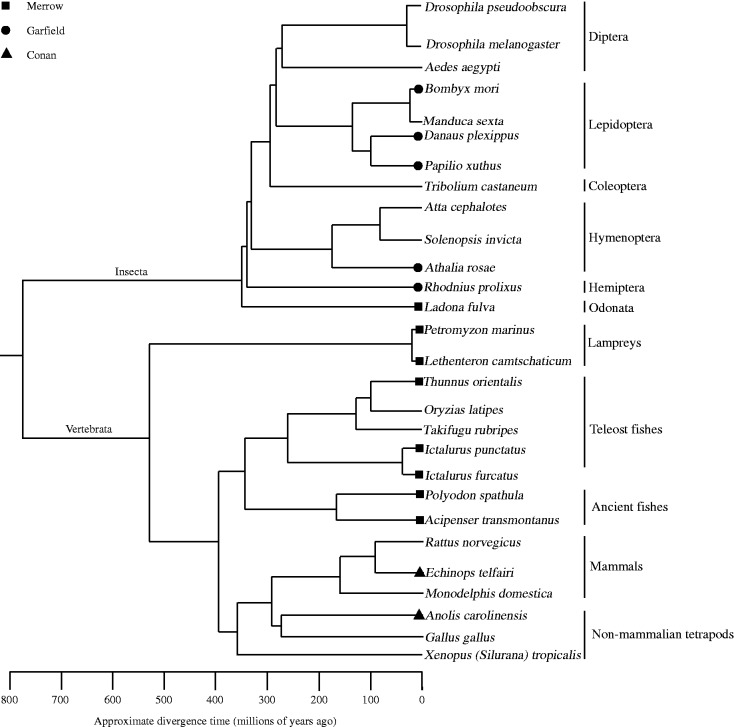
Schematic representation of a phylogenetic tree of animal lineages, estimated divergence times (Ma) and species distribution of *Merrow*, *Garfield*, and *Conan* identified in this study. The inferred HT events of these three different families were denoted by solid square, solid circles, and solid triangle, respectively.

Multiple alignments of *Merrow*, *Garfield*, and *Conan* identified in this study revealed a strikingly high level of interspecific sequence identity (79–99%). Importantly, the level of nucleotide sequence identity is not only limited to coding regions but also extended to noncoding regions of the elements (supplementary fig. S6, Supplementary Material online). In many cases, the level of nucleotide sequence identity of these transposons is unexpectedly high when considering the deep divergence of their host species (fig. 4). For example, *Merrow* identified in lampreys and teleost fishes as well as dragonfly shared 87–98% pairwise sequence identity. However, lampreys and jawed fishes diverged approximately 500 Ma and they shared a last common ancestor with dragonfly more than 700 Ma (supplementary table S3, Supplementary Material online; Hedges et al*.* 2006). A similarly elevated level of sequence identity (87–99%) of *Garfield* and *Conan* identified in different insect orders (Lepidoptera, Hymenoptera, and Hemiptera), insect viruses, and vertebrates was also observed (Tables S5 and S6). These insect orders diverged from each other more than 300 Ma (Hedges et al*.* 2006). In addition, tenrec and lizard diverged from a common ancestor approximately 300 Ma (fig. 4; Hedges et al*.* 2006). Thus, the extreme level of sequence similarity of *Merrow*, *Garfield*, and *Conan* across such distant species strongly suggests that these transposons invaded their hosts through repeated HT events.

To obtain additional evidence supporting HT, we investigated whether some ancestral copies of *Merrow*, *Garfield*, and *Conan* could be found at orthologous genomic positions in those species in which they were identified. The results showed that none of these transposons was present at orthologous positions in the species studied, with the exception of Arctic lamprey and sea lamprey, where full-length *Merrow* transposons were found at orthologous positions and therefore must have inserted before the divergence of these lampreys (supplementary fig. S7, Supplementary Material online). Next, we examined the taxonomic distribution of these three transposons and found that it was highly discontinuous and inconsistent with the phylogeny of their host species. For example, *Garfield* was only identified in turnip sawfly, but it was undetectable in the genome of 10 other species of hymenopteran insects (Zhang et al*.* 2013). Similarly, *Conan* was present in the tenrec (an afrotherian mammal) and the anole lizard, but it was not found in any of the dozens of other mammalian and reptilian genomes currently available in the databases.

Several additional lines of evidence rule out the possibility that *Merrow*, *Garfield*, and *Conan* were vertically inherited from the last common ancestor of these species. First, the topology of the phylogenetic tree of *Merrow*, *Garfield*, and *Conan* is incongruent with that of the host species (fig. 1 and supplementary fig. S8, Supplementary Material online). Furthermore, *Merrow* identified in closely related teleost fishes showed higher level of nucleotide sequence divergence than those from lampreys and teleost fishes, which diverged approximately 500 Ma (supplementary table S4, Supplementary Material online). Similarly, *Garfield* identified within insects of Lepidoptera exhibited higher sequence divergence at the nucleotide level than *Garfield* transposons from Lepidoptera and other insect orders (Hymenoptera and Hemiptera) (supplementary table S5, Supplementary Material online). In addition, we found no evidence that purifying selection and codon bias could account for the high level of conservation of *Merrow*, *Garfield*, and *Conan* identified in such widely divergent species. Phylogenetic analysis of these transposons obtained from each species showed a star-like shape, an indicative of a single rapid amplification from one master element followed by the accumulation of discrete mutations in each copy (supplementary fig. S9, Supplementary Material online). This evolutionary pattern is consistent with the neutral evolution typical of DNA transposons (Hartl et al. 1997; Feschotte and Pritham 2007). For all autonomous *Merrow* and *Garfield*, we found that the level of synonymous divergence (d*s*) between species was considerably lower than that expected between such highly diverged taxa. For example, lampreys and jawed fishes separated more than 500 Ma (Hedges et al*.* 2006), yet the d*s* between lampreys and the white sturgeon *Acipenser transmontanus Merrow* consensus was 0.0088 (supplementary table S7, Supplementary Material online). Similarly, the d*s* values between *Garfield* consensus sequences of insects were all lower than 0.0718. Importantly, d*n*/d*s* for *Merrow* and *Garfield* varied from 0.2632 to 1.3068, consistent with low to no significant purifying selection acting on these transposons (supplementary table S7, Supplementary Material online). Furthermore, d*s* values for a well conserved housekeeping gene such as the elongation factor gene *EF-1α* were at least ten times higher (from 0.5696 to 3.4936) than those based on the sequences of the corresponding *Garfield* (from 0.0092 to 0.0718) (Tables S7 and S8). Together, these data suggested that strong purifying selection was not responsible for the high level of sequence identity of these transposons (at least for *Garfield*) across these widely diverged species. Codon bias as determined by the effective number of codon (*N*_c_) value is known to represent a potential source of selective constraint on synonymous nucleotides (Wright 1990). *N*_c_ values varied from 21 (one codon per aa—high bias) to 61 (all codons used equally—no bias) (Wright 1990). *N*_c_ values for all transposons identified in this study was 49–59 (supplementary table S9, Supplementary Material online), suggesting that codon bias was also not responsible for the observed high sequence identity. Finally, an inferred insertion period of *Merrow*, *Garfield*, and *Conan* postdated the radiation of two species where these transposons resided (supplementary fig. S5, Supplementary Material online). The only exception to this pattern was the inferred amplification time (10–17 Ma) of *Merrow* in lampreys, which fell within the divergence time between these two species (10–30 Ma; Kuraku and Kuratani 2006), and was consistent with the findings that *Merrow* elements occupy orthologous positions in the two lampreys (see above and supplementary fig. S7, Supplementary Material online). Interestingly, our dating (26–36 Ma) for the invasion of *Conan_ET* in the tenrec fell within the range inferred for the HT cases previously reported for several *hAT* transposons in diverse tetrapods, including the tenrec (15–46 Ma; Pace et al*.* 2008; Gilbert et al*.* 2010). Tenrecs are confined to Africa (Poux et al*.* 2005) and the anole lizard (which acquired a nearly identical *Conan* element) most likely has been endemic to South America (Roughgarden 1995). As the African and American continents separated much earlier (>65 Ma; Marshall et al*.* 1979) than the inferred introduction of *Conan* in the tenrec lineage, these observations suggest that *Conan* underwent a transoceanic movement, similarly to and around the same time as other widely horizontally transferred transposons (Pace et al. 2008; Gilbert et al. 2010). Interestingly, the *Garfield* element identified in *Cotesia sesamiae Mombasa bracovirus* (EF710639) was seemingly full-length and capable to encode an apparently intact Tpase (fig. 3), suggesting that it might represent a recent acquisition by this insect virus.

Together, these data indicate that the most plausible scenario to explain the distribution of *Merrow*, *Garfield*, and *Conan* examined in this study is that these transposons were transferred horizontally into multiple species lineages and subsequently expanded within each genome. HT events between insects and insect bracoviruses (Thomas et al*.* 2010), between lampreys and teleost fishes (Kuraku et al*.* 2012), and between the tenrec and lizard (Pace et al. 2008; Gilbert et al. 2010) have been previously described. Thus, we speculate that these taxa have a higher propensity for exchanging genetic material.

We also note that HT of *Conan* between tenrec and lizard has been alluded to in Repbase Reports (Kapitonov and Jurka 2007). However, to our knowledge HTs of *Merrow* and *Garfield* have not been reported previously. The clear phylogenetic separation of these three families of *Chapaev* transposons (fig. 1) indicates that these three families have been independently transferred into multiple hosts.

### Possible Vectors and Factors Facilitating HT

*Merrow*, *Garfield*, and *Conan* were identified in such a wide range of species (including not only lampreys, jawed fishes, lizard, tenrec but also silkworm, two distant butterflies, turnip sawfly, triatomine bug, dragonfly, and a bracovirus), suggesting that multiple vectors and mechanisms might be involved in the HTs of these transposons. *Garfield* discovered in the bracovirus was of particular interest because bracoviruses might represent an ideal vector for the horizontal spread of these transposons among species. These viruses create an obligatory relationship with parasitic wasps, and they only replicate in the ovary cells of wasps. Then, fully formed viral particles in the wasp ovary are injected into the lepidopteran larvae by the wasps. Thus, the intimate association between the parasitoid and their lepidopteran hosts might provide ample opportunity for the HTs of transposons. Indeed, there have been several documented examples of exchanging genetic materials between DNA viruses and their insect hosts (Fleming and Summers 1991; Jehle et al. 1998; Marquez and Pritham 2010; Thomas et al. 2010; Dupuy et al. 2011; Gilbert et al. 2014).

Another interesting finding was the identification of nearly identical *Merrow* in lampreys and teleost fishes (table 1). Lampreys are opportunistic parasitic feeders that attach to large fish using their cup-like mouth to suck their blood and body fluids. The exchange of large amounts of blood between lampreys and their fish hosts during this parasitic interaction might provide a potential route for the horizontal spread of transposons, as suggested previously for *Tc1*-like transposons (Kuraku et al. 2012). Interestingly, lampreys are known to commonly parasitize sturgeons (Patrick et al*.* 2009) and paddlefish (Hardisty and Potter 1971), two species that harbor *Merrow* elements nearly identical to those of lampreys (table 1).

## Supplementary Material

Supplementary tables S1–S9 and figures S1–S9 are available at *Genome Biology and Evolution* online (http://www.gbe.oxfordjournals.org/).

Supplementary Data
