# Supplementary material for: Recurrent Horizontal Transfers of Chapaev Transposons in Diverse Invertebrate and Vertebrate Animals
Source: Genome Biol Evol. 2014 May 27;6(6):1375–86. doi: 10.1093/gbe/evu112 (PMC4079192; doi:10.1093/gbe/evu112)
Supplement: Supplementary Data [file supp_6_6_1375__index.html]

Recurrent horizontal transfers of Chapaev transposons in diverse invertebrate and vertebrate animals — Recurrent Horizontal Transfers of Chapaev Transposons in Diverse Invertebrate and Vertebrate Animals — Supplementary Data 

# Recurrent Horizontal Transfers of *Chapaev* Transposons in Diverse Invertebrate and Vertebrate Animals

## Supplementary Data

files

**Files in this Data Supplement:**

- Supplementary Data - pdf file
