# Supplementary material for: Recurrent Horizontal Transfers of Chapaev Transposons in Diverse Invertebrate and Vertebrate Animals
Source: Genome Biol Evol. 2014 May 27;6(6):1375–86. doi: 10.1093/gbe/evu112 (PMC4079192; doi:10.1093/gbe/evu112)
Supplement: Supplementary Data [file supp_evu112_SupportingInformationR2.pdf]

```
1     ATGTCGTCACGAAAGTGCAAATACGATGCTGATGCATTTTGTCTTATATGTGGTCAATTTATTAAAGTTCGAGACGTGAAATATGAACTAAAGACATCTCACGTTCTCTGTGAAGCCTAT
       M  S  S  R  K  C  K  Y  D  A  D  A  F  C  L  I  C  G  Q  F  I  K  V  R  D  V  K  Y  E  L  K  T  S  H  V  L  C  E  A  Y   40
121   GAAGCATATTTTGACTGTCCTGTACGGAATCAAGATAAGCCATGGGCTCCACATGTTGCTTGTAGTTATTGTAAAAGGTGTTTGGAAGGTTGGTATCGAGGTGAGAAAAGGTCTATGAAA
       E  A  Y  F  D  C  P  V  R  N  Q  D  K  P  W  A  P  H  V  A  C  S  Y  C  K  R  C  L  E  G  W  Y  R  G  E  K  R  S  M  K   80
241   TTTGCAATACCAAGGATTTGGCGAGAACCAAAAGACCATATTACTGACTGCTACTTTTGTATGGTGAATCCGAGTAAAAGACGTAGAGGTAAAAATGCAAAATCTATTGAATATCCTGAC
       F  A  I  P  R  I  W  R  E  P  K  D  H  I  T  D  C  Y  F  C  M  V  N  P  S  K  R  R  R  G  K  N  A  K  S  I  E  Y  P  D   120
361   CTCGAATCTTCTTCTGCTCCAATTGCTCACGACCTGACACGACCAGTACCTGAGCCACCAAAAAAATTATCGCAGAAAAGTAGCTCATCTTTTAGTTCTTATAAAAGTAATTCCGATAAG
       L  E  S  S  S  A  P  I  A  H  D  L  T  R  P  V  P  E  P  P  K  K  L  S  Q  K  S  S  S  S  F  S  S  Y  K  S  N  S  D  K   160
481   GAGTTTTTGACTACACCTGAACAACCAAAACACTATCTCATTACTTCAGAAGATTTTAACGATCTAATTAGAGATTTAAATTTGCCAAAAAATAAAGCAGAGCTTCTAGGCTCTCGGTTA
       E  F  L  T  T  P  E  Q  P  K  H  Y  L  I  T  S  E  D  F  N  D  L  I  R  D  L  N  L  P  K  N  K  A  E  L  L  G  S  R  L   200
601   AAACAGTGGAATTTGCTTGATGATGTTAAGATCACGGATCAGCGGACTAGGCATGAAATGTTTGCAACGTTTTTCACGAAGGAAGATGGACTTTGTTTTTGTAATGACATTAAAGGTATG
       K  Q  W  N  L  L  D  D  V  K  I  T  D  Q  R  T  R  H  E  M  F  A  T  F  F  T  K  E  D  G  L  C  F  C  N  D  I  K  G  M   240
721   TTTGAAGCAATTGGCATACCCTGTGTCCCTAGCGAATGGCGTTTATTCATTGACAGCTCTACAAAAAGTTTAAAAGCAGTTCTATTGCACAACAGAAATAAATTTCCGTCTCTTCCAATT
       F  E  A  I  G  I  P  C  V  P  S  E  W  R  L  F  I  D  S  S  T  K  S  L  K  A  V  L  L  H  N  R  N  K  F  P  S  L  P  I   280
841   GCTCACTCAGTACATCTTAAAGAAAATTATGAAAGTGTCAAAATTTTGCTTGAATGTGTAAAATATCGTGAGTATAACTGGGAGCTGATTGGAGATTTTAAAATGGTGGGATTTTTAATG
       A  H  S  V  H  L  K  E  N  Y  E  S  V  K  I  L  L  E  C  V  K  Y  R  E  Y  N  W  E  L  I  G  D  F  K  M  V  G  F  L  M   320
961   GGGCTACAGGGTGGATATACAAAGTATCCGTGTTATTTGTGCTTGTGGGATAGCAGAGCGGATTCTAAACACTATATTCAACGGTCATGGCCTGTAAGAACAGAATTGTGTGTCGGAAAA
       G  L  Q  G  G  Y  T  K  Y  P  C  Y  L  C  L  W  D  S  R  A  D  S  K  H  Y  I  Q  R  S  W  P  V  R  T  E  L  C  V  G  K   360
1081  CAGAACGTCAAATTCGAGCCGATTGTTGAAGCGGAAAAAGTGTTAATGCCGCCTTTGCACATTAAGTTAGGGTTTATGAAACAATTTGTTAAAAAACTGGATGAAACTTCAGAAGCTTTT
       Q  N  V  K  F  E  P  I  V  E  A  E  K  V  L  M  P  P  L  H  I  K  L  G  F  M  K  Q  F  V  K  K  L  D  E  T  S  E  A  F   400
1201  GGATACTTAAAAAATTTTTTTCCGAAGTTATCGGAAGCAAAGGTTAAAGCTGGGGTTTTTGTTGGTCCGCAAATAAGACAGATTTTCGCCGATGAAAAATTTCCAACGTTGCTGAATCGT
       G  Y  L  K  N  F  F  P  K  L  S  E  A  K  V  K  A  G  V  F  V  G  P  Q  I  R  Q  I  F  A  D  E  K  F  P  T  L  L  N  R   440
1321  ACTCAAAAAGCAAGTTGGAACAGTTTTAAAGCAGTAGTTTCTGGATTTTTAGGAAATAATAAAGCTGAAAACTACGAAAAGTTGGTTGAGGATATGCTTACAAATTTTAAGGCCATGGGT
       T  Q  K  A  S  W  N  S  F  K  A  V  V  S  G  F  L  G  N  N  K  A  E  N  Y  E  K  L  V  E  D  M  L  T  N  F  K  A  M  G   480
1441  TGCAGGATGTCATTAAAAGTACATATGCTGCATGCTCATTTGGATAAATTTAAAAACAATATGGGAGCCTATTCTGAAGAGCAAGGACAACGTTTCCATCAGGACATCATGAATTTTGAA
       C  R  M  S  L  K  V  H  M  L  H  A  H  L  D  K  F  K  N  N  M  G  A  Y  S  E  E  Q  G  Q  R  F  H  Q  D  I  M  N  F  E   520
1561  CAACGCTATCAAGGCCAATACAATGAAAACATGATGAGCGACTATATTTGGGGTTTATTGAGAGAAAGTAGCTATGAACATAAAAGAAAAAGTAAAAGTGTGCACTTTTAA
       Q  R  Y  Q  G  Q  Y  N  E  N  M  M  S  D  Y  I  W  G  L  L  R  E  S  S  Y  E  H  K  R  K  S  K  S  V  H  F  *               556
```

Figure S1: Nucleotide sequence and amino acid translation of the coding region of *Garfield_BM*. The three conserved motifs (C(2)C, LH, and H(4)H) encoded by its transposase were shown using black underline. Its first exon was also shown using black underline. Stop codon was indicated using star.

Garfield_BM

```
nscaf2825_492730_494338_+  : GTTCTCTATCAGACCTCAAACTGCCGGAATCTATAGACA----AATTACACAACTCTACAAAAAAAA-------------------TCAAATTTGGTGTTGACTCGTGTTATCTCT-TAACAGACTAAAAGGGCGTTTTCTG
nscaf463_1608556_1610353_+ : ------ATATAGTCTCGAAATT----------------ATGACAATAACACGACTCTACAAAAAAAT-------------------TCAAATTTCATGTTGACCCGTGTAATCTATACAGCATTGTAATGATACCTTTCCTA
nscaf3031_2217815_2219367_-: -----AAAGCTTACTATAAACT-----------------CAATGATTACACGACTCTACAAAAAAAT-------------------TCAAATTTCATGTTGACCCGTGTTAT-----CTAGAAGAT-----TGCACAAAGTG
nscaf2938_33590_35561_-    : CCATCAAAGCAA--TAAAAATAAATAAAGAAACGAGCCATAT--ATAACACGACTCTACAAAAAAAT-------------------TCAAATTTCATGTTGACCCGTGTAATTTATGTCATAGA--------ACATGATCGA
nscaf2779_65700_67158_+    : ------AAACAGGTTGCAAACTGTCG--------TGTCTTAATAATAACACGACTCTACAAAAAAAA-------------------TCAAATTTCATGTTGACCCGTGTAATTATCACCACATAAT-ACCACACACGCTCTA
nscaf2948_15381_17812_-    : TCTACAAGATAGCATGTACATTTATGAAAAATT-AGTTATATAAATTACACGACTCTACAAAAAAAT-------------------TCAAATTTCATGTTGACCCGTGTTATCTTAACTAGAATCTTAACTATCCAGTTTTA
```

Merrow_PM

```
gb|AEFG01003823.1|_3490_6037_+   : AACACAAATATGAATGTTCATGTAGCAATAGAAT----------GATTACACTGTGTAACCA-TTTT---------------------AAAAAATGTGTTACATAGTGTTATCTT---------TGTTTCCAGGAACTTGGC
gb|AEFG01029931.1|_1032_3714_+   : ATAATAGTGGTGAGT-----------------------------GATTACACTGTGTAACAA-TTTT---------------------AAAAATTGTGTTACATAGTGTTATCTG-------------------AAGCAGTC
gb|AEFG01026769.1|_13752_15924_- : GAGAAATAGTTAACCATCTCTGAACTAATGGTAGAGT-------CATTACACTGTGTAACAATTTTT---------------------AACAATTGTGTCACATAGTGTTATAAA---------AAAACCATTGGGAATTAA
gb|AEFG01002365.1|_11396_14039_+ : AATGATTGCGCAACAAAACTCCCCTTT-----------------GATAACACTGTATAACAA-TTTT---------------------AAAAATTGTGTTACGTAGTGTAATTAG--------TTTACTGCATTTGAATGCT
gb|AEFG01037147.1|_5677_8280_+   : GCAAACTTTATAGTTA----------------------------AATTACACTGTGTACCAA-TTTT-----------------------AAATTGTGTTACATAGTGTTATTTATGGGGAAATGTTTTTGCTTAACATTCC
gb|AEFG01005055.1|_26341_28970_+ : TAGCGCGCTGCACTACGAACCCCACTTACAACAACACCGGCGACGATTACACTGTGTAACATTTTTT---------------------AAAAATTGTGTTACATAGTGTTATCAA---------CTCAGCCTCAAATGAGTA
gb|AEFG01016281.1|_22093_24730_+ : TGGTGAGGTGTGATT-----------------------------AATAACACTGTGTAACAT-TTTT---------------------AAAAATTTTGTTACATAGTGTAATCAA----TCATCATAACCCCTATGACCCTC
gb|AEFG01010003.1|_6220_8852_-   : TTCATAACTGAAGTCTCAATTCAAG-------------------AATAACACTGTGTAACAT-TTTT---------------------AAAAATTGTGTTACATAGTGTAATCTA-------------------CACTGACA
```

Figure S2: Insertion bias of *Garfield_BM* and *Merrow_PM*. Their TSD were showed using red color, and an "A" on the 5' end and a "T" on the 3' end were indicated using blue color. Species abbreviations: PM, *Petromyzon marinus*; BM, *Bombyx mori*.

```
gb|APJL01060134.1|_12301_14910_+ : TCCCACTCATGGCCAACACCAGTGACGATTA  (Merrow_LC)  TTATCTGAGCCAGTCATGGGCCTCCCCTAGGTAAACTCAACCTCAAAATGAGT
gb|APJL01135482.1|_1359_1481_+   : TCCCACTCACGGCCAACACCAGTGACGATTA---------------TCTGAACCGGTCTTGGGCCTCCTTTAGGTCGACTCAGCCTCAAAATTAGT
gb|APJL01089792.1|_5626_5750_-   : TCCCGCTCATGGCCAACACCAGTGACGATTA---------------TCTGAACCGGTCCTGAGCCTCCCCTGGGTCAACTCAGCCTC-AAATGAGT
gb|APJL01085885.1|_3324_3447_-   : TCCCGCTCACGGCCAACACGAGTGCCGATTA---------------TCTGAACCGGTCCTGGGCCTCCCCTAGGTCGACTCGGCCTC-AAATGAGT
gb|APJL01175379.1|_467_594_+     : TCCCGCTCACGGCCAACACCAGTGACGATTA---------------TCTGAACCGGTCCTGGGCCTCCCCTAGGTCGACTCAGCCTCAAAATGAGT
gb|APJL01042531.1|_1759_1885_-   : TCCCGCTCATGGCCAACACCAGTGATGATTA---------------TCTGAACCGGTCCTGGGCCTCCCCTAGGTAGACTCAGCCTC-AAATGAGT
gb|APJL01107956.1|_231_357_+     : TCCCGCTCACGGCCAACACCAGTGACGATTA---------------TCTGAACCGGTCATGGGCCTCCCCTAGGTCGACTCAGCCTC-AAATGAGT
gb|APJL01115088.1|_410_536_-     : TCCCTCTCACGGCCAACACCAGTGACGATTA---------------TCTGAACCGGTCCTGGGCCTCCCCTAGGTCGACTCAGCCTC-AAATGAGT
gb|APJL01144824.1|_570_696_-     : TCCCGCTCACGGCCAACACCAGTGACGATTA---------------TCTGAACCGGTCCTGGGCCTCCCCTAGGTAGACTCAGCCTC-AAATGAGT
```

```
nscaf3031_2217816_2219367_+ : TCATTCCCACTTTGTGCAATCTTCTAGATAA  (Garfield_BM)  TAATCATTGAGTTTATAGTAAGCTTTATTGCACAGATGTTCTTTAATAGTTTT
nscaf2851_26418_26586_+     : TCATTCCCACTTTGTGCAATCTTCTAGATAA--------------- TCATTGACTTTATAGTACACTTTATGGCACAGATGTTCTTTAATAGTTTT
nscaf3079_1299454_1299622_- : TCATTCCCACTTTGTGCAATCTTCTAGATAA--------------- AAATTGATTTTATAGTAAGCTTTATTGCACAGATATTCTTTGATAGTCTT
nscaf2993_1383358_1383526_- : TCTTTCCCCCTTTGTGCAATCTTCTAGATAA--------------- TCATTGACTTTATAGTGAGCTTTATTGCACAGATGTTCTTTAATAGTTTT
nscaf3058_7739461_7739629_+ : TCATTCCCACTTTGTGCAATCTTCTAGATAA--------------- TCATTGACTTTATAGTAAGCTTTATTGCACAGATGTTCTTTAATAGTTTT
nscaf531_2138_2306_-        : TCATTCCCACTTTGTGCAATCTTCTAGATAA--------------- TCATTGACTTTATAGTAAGCTTTATTGCACAGATGTTCTTTAATAGTTTT
nscaf2930_4483339_4483507_  : TCATTCCCACTTTGTGCAATCTTCTAGATAA--------------- TCATTGACTTTATAGTAAGCTTTATTGCACAGATGTTCTTTAATAGTTTT
nscaf3003_1144877_1145045_+ : TCATTCCCACTTTGTGCAATCTTCTAGATAA--------------- TCATTGACTTTATAGTAAGCTTTATTGCACAGATGTTCTTTAATAGTTTT
nscaf3022_57806_57974_+     : TCATCCCCACTTTGTGCAATCTTCTAGATAA--------------- TCATTGACTTTATAGTAAGCTTTATTGCACAGATGTTCTTTAATAGTTTT
```

Figure S3: Paralogous "empty" site of *Merrow_LC* and *Garfield_BM*. Multiple alignments indicating the presence or absent of *Chapaev3* elements at paralogous loci within the silkworm and Arctic lamprey. Their TSD were showed using underline. Species abbreviations: LC, *Lethentero camtschaticum*; BM, *Bombyx mori*.

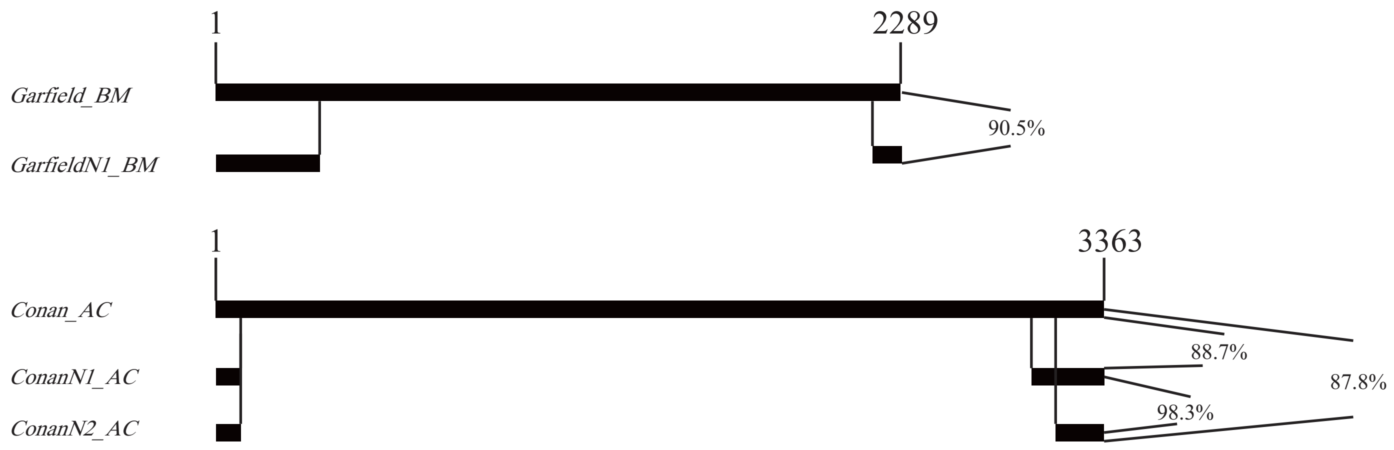

Figure S4: Schematic of regions of shared identity between autonomous *Garfield_BM* and *Conan_AC* transposons and their derived MITEs in the silkworm and lizard. Percentages of identity were calculated using the software Bioedit (HALL 1999). Species abbreviations: BM, *Bombyx mori*; AC, *Anolis carolinensis*.

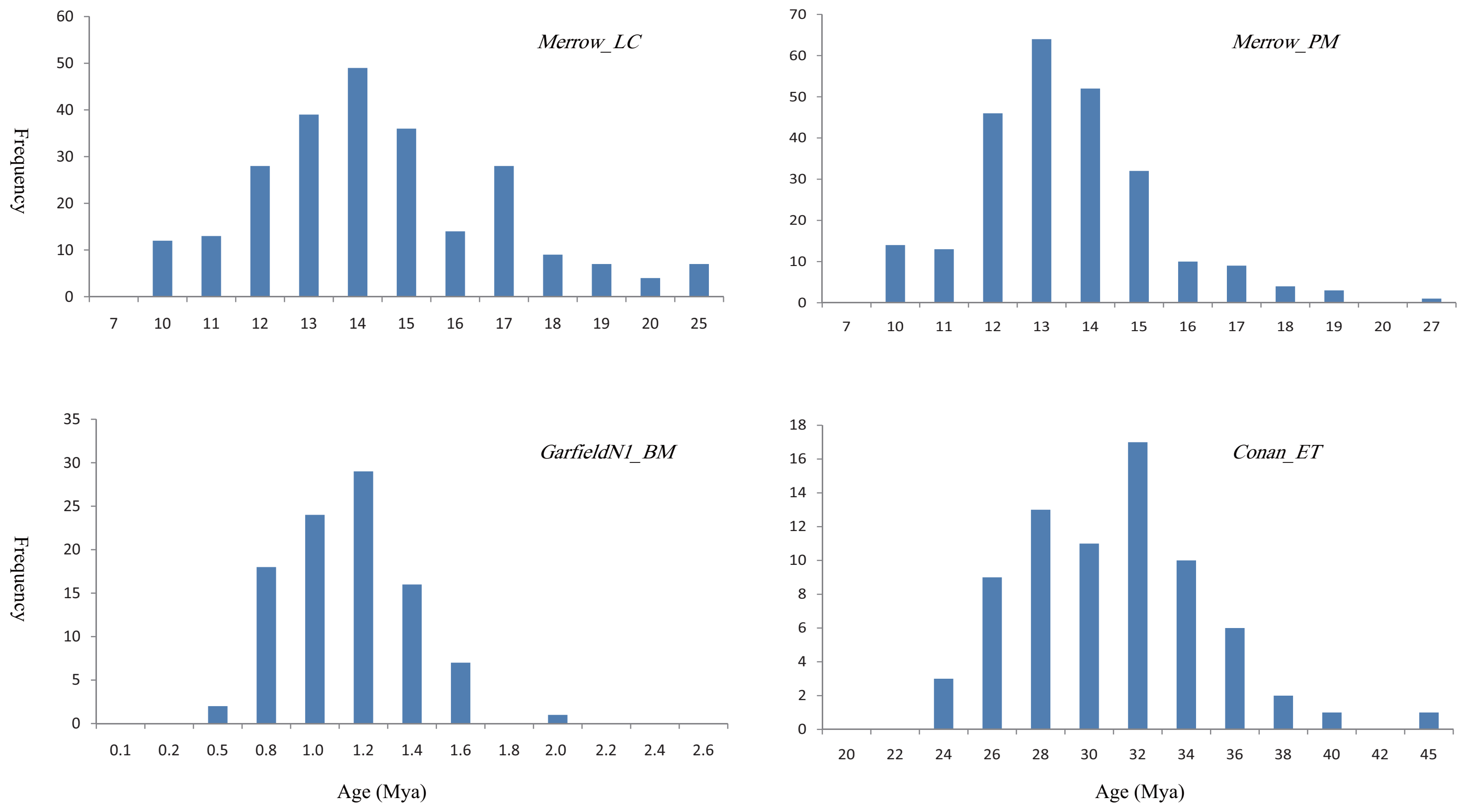

Figure S5: An inferred timing of *Merrow_LC*, *Merrow_PM*, *Garfield_BM* and *Conan_ET*. Because there is no reliable neutral mutation rate available for other species or their close related taxa, these species were not included in this analysis. Species abbreviations: LC, *Lethenteron camtschaticum*; PM, *Petromyzon marinus*; BM, *Bombyx mori*; ET, *Echinops telfairi*.

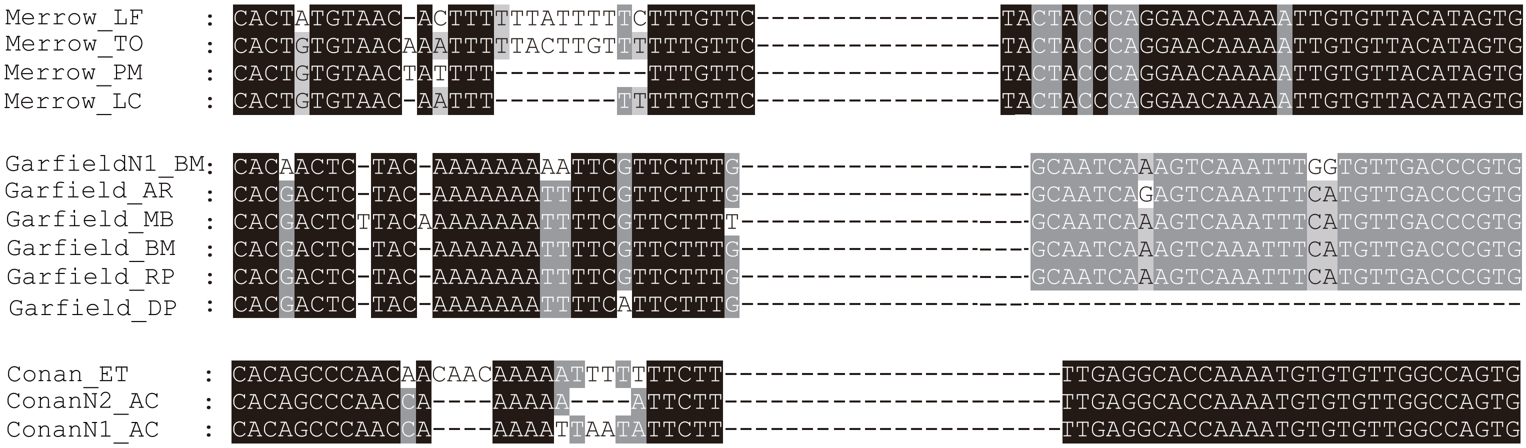

Figure S6: Multiple alignment of *Merrow*, *Garfield* and *Conan* identified in this study, showing portions of the highly conserved 5′ and 3′ termini (black). Species abbreviations: LF, *Ladona fulva*; TO, *Thunnus orientalis*; PM, *Petromyzon marinus*; LC, *Lethenteron camtschaticum*; BM, *Bombyx mori*; MB, *Cotesia sesamiae Mombasa bracovirus*; AR, *Athalia rosae*; RP, *Rhodnius prolixus*; DP, *Danaus plexippus*; AC, *Anolis carolinensis*; ET, *Echinops telfairi*.

```
gb|APJL01056192.1|_7077_9679_-      : GCGACTTAAGTTTAATTTCCCGGCAATGGTGAATGCACTGTGTAACGATTT--   Merrow_LC   --GAAAATTGTGTTACATAGTGATGTCAGTGGAAAGTGGTATCGAAGCT
gb|AEFG01000174.1|_8471_11097_-     : GCGACTTAAGTTTAATTCCCCGACAATGGTGAATGCACTGTGTAACAATTT--   Merrow_PM   --GAAAATTGTGTTACATAGTGATGTCAGTGGAAAGTAGTATCGAAGCT

gb|APJL01000384.1|_586_3199_+       : TACTTTTAGACGTTGAAGGTTTTTTTTCTTCATAACACTATGTAACATTTG--   Merrow_LC   --AAAAATTGTGTTACATAGTGTAATTAGTTGTCTGTGTTTACTCAAGG
gb|AEFG01071081.1|_20140_22751_+    : TACTTTTAGACGTTGAAGGGTTTTTTT-TTCTTAACACTGTGTAACAAATT--   Merrow_PM   --AAAAATTGTGTTACATAGTGTAATTAGTTATCTGTGTTTACTCAATG

gb|APJL01012584.1|_27143_29740_-    : TCACACTGACCAGCATAG TGAGGTGTGATCAATAACACTGTGTAACAATTA--   Merrow_LC   --AAAAAATGTGTTACATAGTGTAATCAATCATCATAACCCCCATAACC
gb|AEFG01016281.1|_22093_24730_+    : CCACACTGACTAGCATGG TGAGGTGTGATTAATAACACTGTGTAACATTT---   Merrow_PM   --AAAAATTTTGTTACATAGTGTAATCAATCATCATAACCCCTATGACC

gb|APJL01008952.1|_3361_5981_-      : GCAGACATTCATTGGAAACAGTACTTTAGTGATATCACTGTGTAACAATTT--   Merrow_LC   --AAAATTTGTGTTACATAGTGTATTCATTCAAAATGTATCAAGATTAA
gb|AEFG01003711.1|_34568_37172_+    : GCAGACACTCATTGTAAACAGTACTTTAGTGATATCACTGTGTAACAATTT--   Merrow_PM   --AAAATTTGTGTTACATAGTGTATTCATTCAGAATGTATCAAGATTAA
```

Figure S7: Four examples of *Merrow* identified at orthologous loci of Arctic lamprey and sea lamprey, suggesting that they must have inserted before the radiation of these lampreys. Species abbreviations: LC, *Lethenteron camtschaticum*; PM, *Petromyzon marinus*.

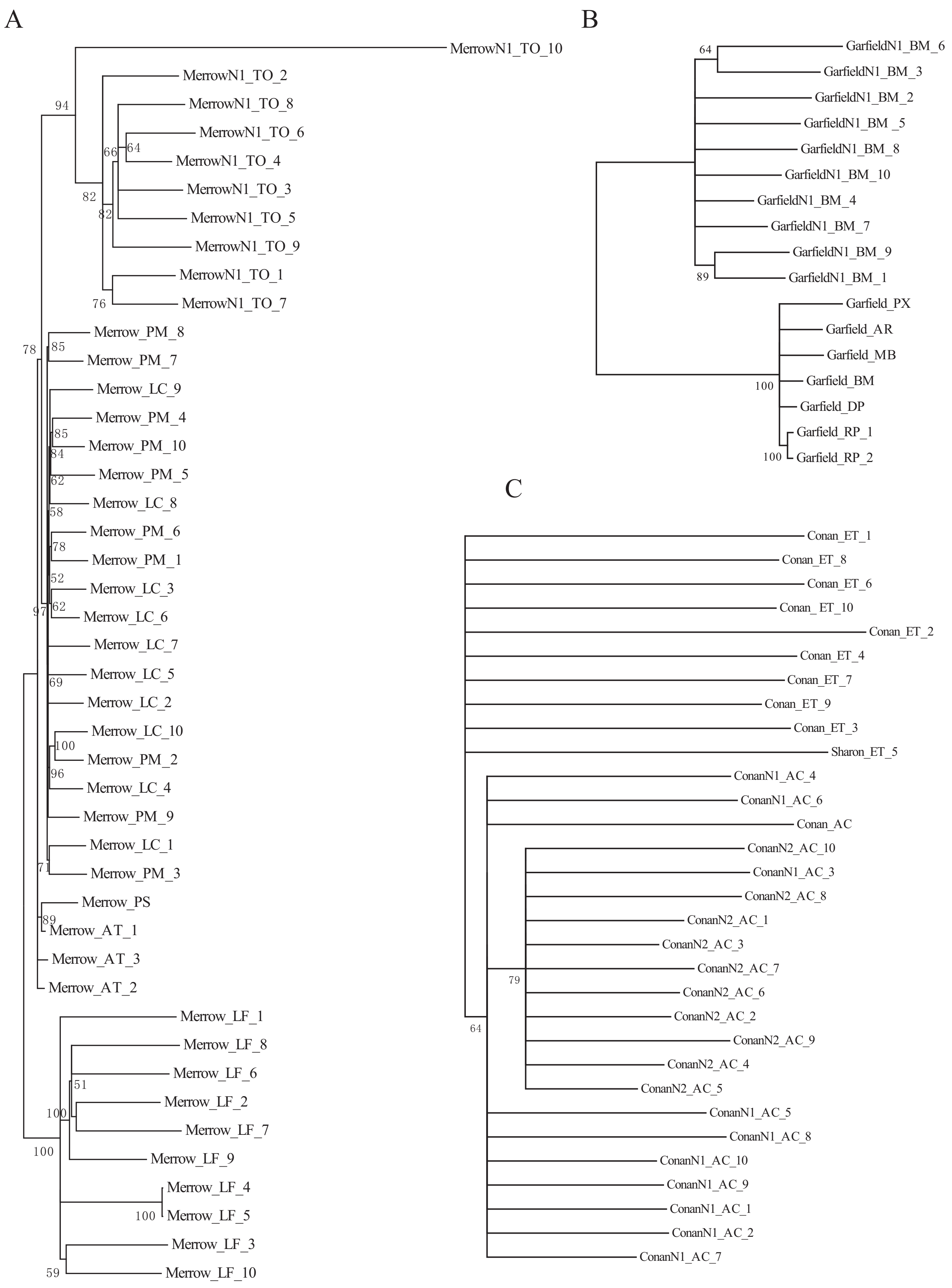

Figure S8: Phylogenetic analysis of *Merrow* (A), *Garfield* (B) and *Conan* (C) based on nucleotide sequences from their copies. Phylogenies were created using MRBAYES 3.1.2 software. Clade credibility values (> 50%) are shown at each node. Species abbreviations: LF, *Ladona fulva*; TO, *Thunnus orientalis*; PM, *Petromyzon marinus*; LC, *Lethenteron camtschaticum*; AT, *Acipenser transmontanus*; BM, *Bombyx mori*; MB, *Cotesia sesamiae Mombasa bracovirus*. AR, *Athalia rosae*; RP, *Rhodnius prolixus*; DP, *Danaus plexippus*; PX, *Papilio xuthus*;AC, *Anolis carolinensis*; ET, *Echinops telfairi*.

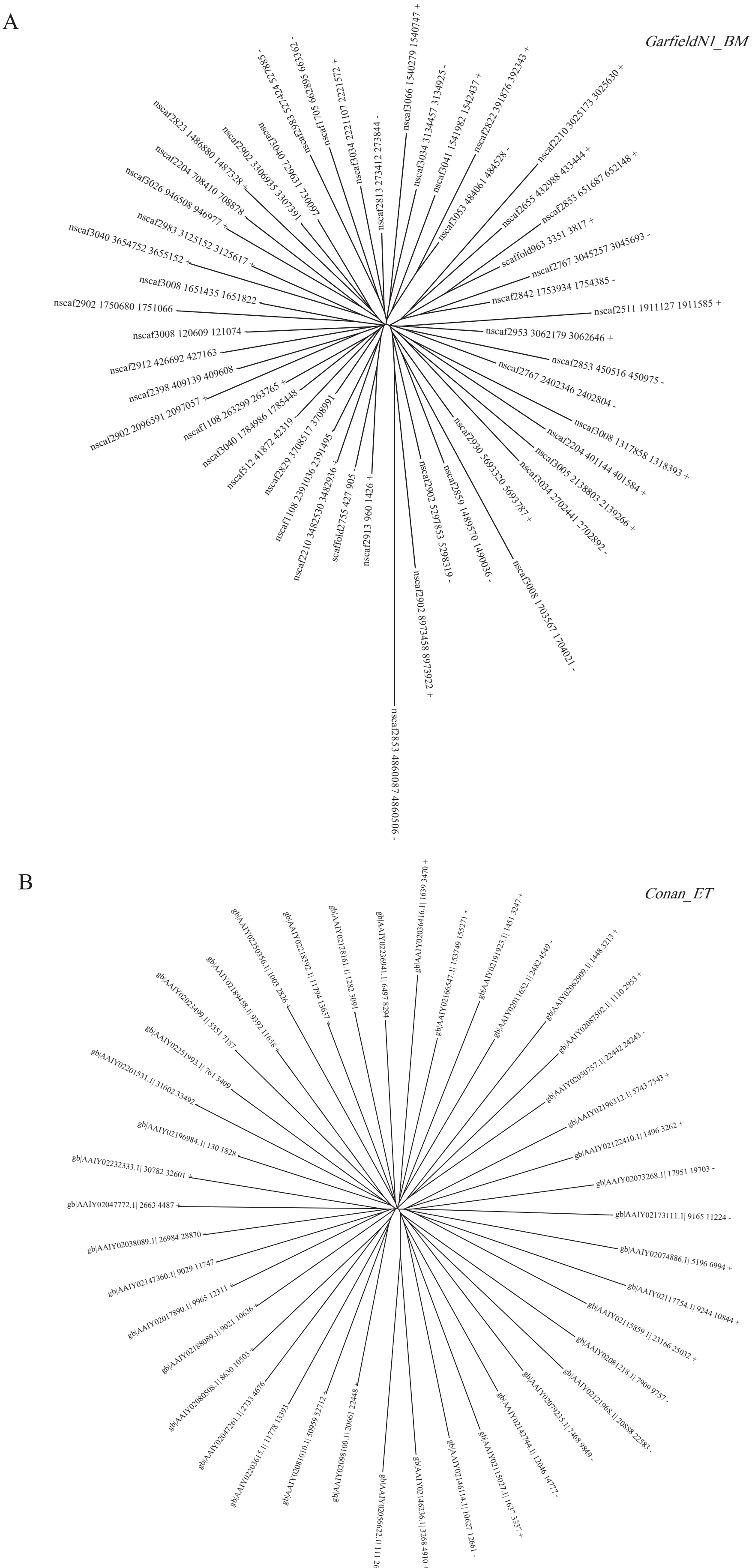

Figure S9: Phylogenetic relationships between members of (A) *GarfieldN1_BM* in the silkworm, (B) *Conan_ET* in tenrec, (C) *ConanN1_AC* and (D) *ConanN2_AC* in lizard. The alignment were built using MUSCLE and ambiguous regions were removed. The trees were created using MEGA4. Phylogenetic analysis of *Garfield* and *Conan* obtained from each species showed a star-like shape indicative of a single rapid amplification from one master element followed by the accumulation of discrete mutations in each copy.

C

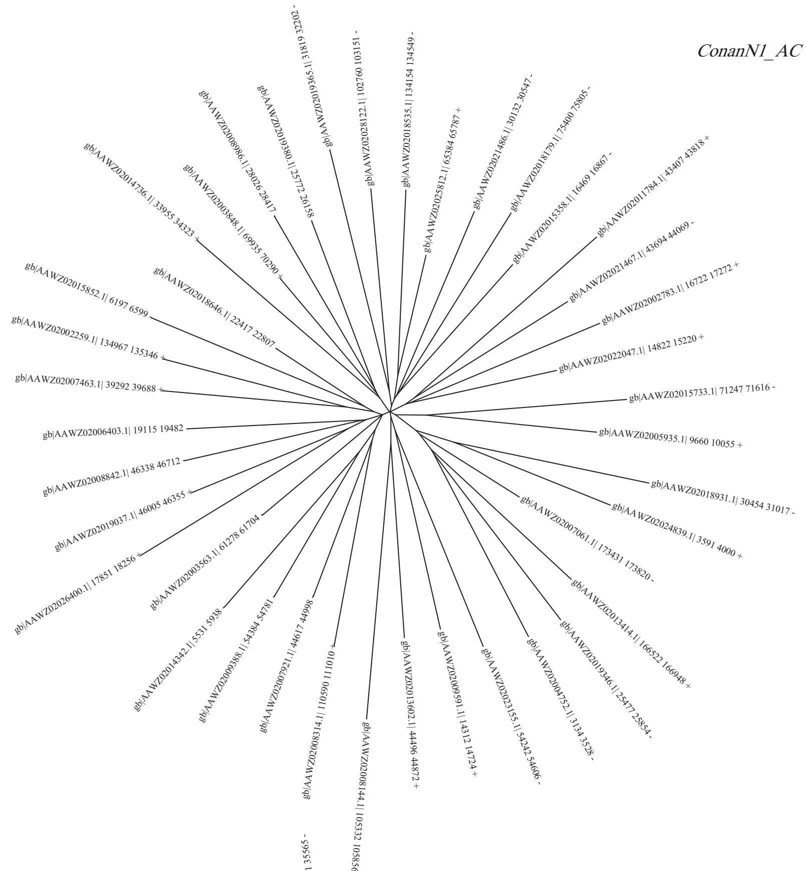

D

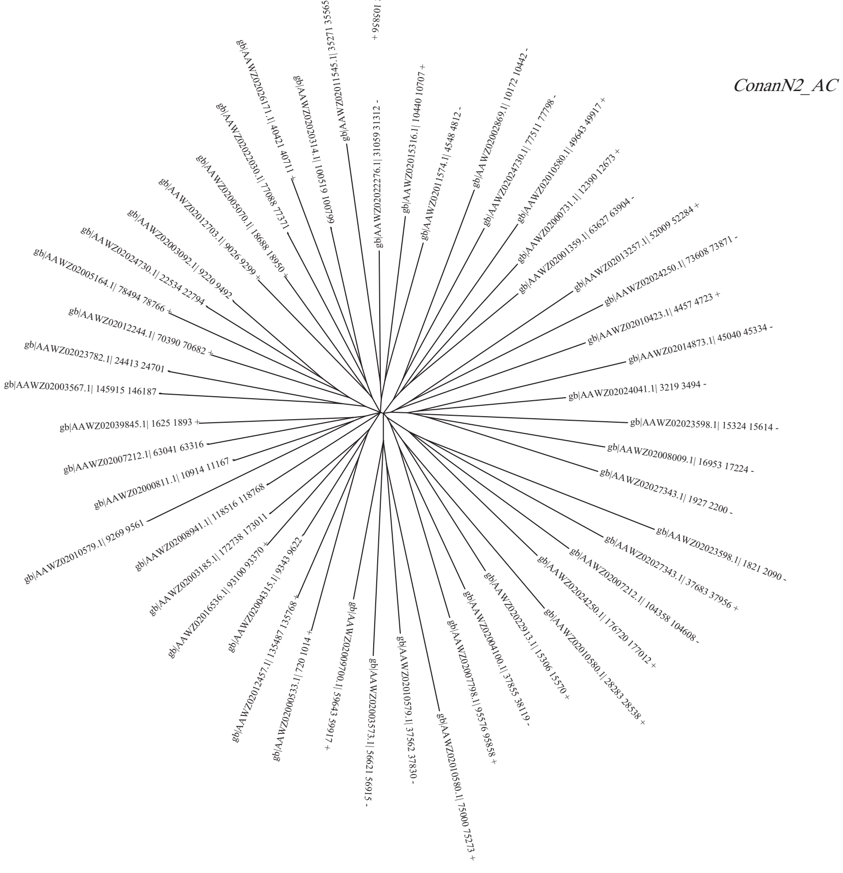

Figure S9 (Continue)

Table S1 Number of copies was used for reconstructing consensus in each species

TE family	Number of copies used for reconstructing consensus
Merrow_LC	50
Merrow_LF	16
MerrowN1_TO	18
Garfield_BM	7
GarfieldN1_BM	50
Garfield_RP	4
ConanN2_AC	50

Table S2 Primers and expected sizes for PCR verification of *Merrow*, *Garfield* and *Conan* identified in this study.

Family	Expected size (bp)	Primer	
		Forward	Reverse
Merrow_LC	1815	GGTTCAAATCCTGGCAGCCATCC	TTGAAATGCGGTCCAGTGCCTTA
MerrowN1_TO	1420	GCACAAAAACCAGGTGTGACAAC	ACCATCCAGCATTATCGTCTTCA
Merrow_IP	593	TGAGAAACCCATATTACCCCAACC	ATGGCTCCTACTTGTCGGTGTTC
GarfieldN1_BM	735	CCGTGGCATACATTAACCAGTAATCAT	CAAGATAGTGTCAACGTCAAACTGC
Garfield_PX	498	GATTGGTGAAGCGGAAAATGTGT	GACGCCCATCATGTTTTCATTGT
Garfield_AR	1111	TGTTGGACTCAGAATGAATAAGCG	CGTGTAGCCAACAATGTGTTTCT
ConanN1_AC	797	ATGGTCATAGAGAAACTCGCTTAG	AAGGTATACATTGCCCACTCTTG
ConanN2_AC	474	GTTTCCATCATCCCTCACTGTTG	TTCTGGTATGCCGAAAGTTGTGG

Species abbreviations: LC, *Lethenteron camtschaticum*; TO, *Thunnus orientalis*; IP, *Ictalurus punctatus*; BM, *Bombyx mori*; PX, *Papilio xuthus*; AR, *Athalia rosae*; AC, *Anolis carolinensis*.

Table S3 Characterization of TSD and nucleotides adjacent to TSD of *Chapaev3* transposons

TE names	Copies	5′				3′				TSD																				
		**A**	T	C	G	A	**T**	C	G	**TWA**	TAG	TTC	TGA	TAC	AAT	ATG	CTA	GTA	ATT	GAA	TCA	TTG	CCA	ATA	CAA	TGG	TTT	CTA	AAA	TAT
Chapaev-20_HM	17	**17**	0	0	0	0	**16**	1	0	**16**	0	0	0	0	0	0	0	0	0	0	0	0	0	0	0	0	0	1	0	0
Chapaev3-4_HM	31	**29**	0	0	2	0	**30**	1	0	**19**	1	0	0	0	0	0	0	0	0	0	0	0	0	1	0	0	2	1	4	3
Chapaev3-5_HM	23	**22**	0	1	0	1	**21**	1	0	**22**	0	0	0	0	0	0	0	0	0	0	0	0	0	0	0	1	0	0	0	0
Chapaev3-6_HM	35	**34**	1	0	0	1	**34**	0	0	**35**	0	0	0	0	0	0	0	0	0	0	0	0	0	0	0	0	0	0	0	0
Chapaev-4_HMa	34	**33**	0	1	0	2	**29**	2	1	**33**	0	0	0	0	0	0	0	0	0	0	0	0	0	0	1	0	0	0	0	0
Chapaev3-1_AC	22	**20**	1	0	1	0	**17**	5	0	**19**	0	1	0	0	0	0	0	0	0	0	0	0	0	0	0	0	1	0	1	0
Chapaev3-2_AC	5	**5**	0	0	0	0	**4**	0	1	**2**	0	0	0	0	0	0	0	0	0	0	0	0	0	0	1	0	1	0	1	0
ConanN1_AC	11	**11**	0	0	0	0	**11**	0	0	**10**	0	0	0	0	0	0	0	0	0	0	0	0	0	0	0	0	0	0	1	0
ConanN2_AC	66	**59**	3	2	2	1	**56**	4	5	**59**	2	0	0	0	0	0	0	0	0	0	1	0	0	0	0	0	0	0	4	0
Merrow_PM	114	**106**	2	1	4	3	**103**	8	0	**103**	1	0	1	0	0	1	0	0	0	0	1	1	1	1	1	0	0	0	3	0
Chapaev3-2_PM	56	**56**	0	0	0	0	**48**	6	2	**53**	0	0	0	0	0	0	0	0	0	0	0	1	0	1	1	0	0	0	0	0
Chapaev-1_BM	15	**14**	0	1	0	1	**12**	1	1	**11**	0	1	0	0	0	0	0	0	0	0	0	1	1	0	0	0	0	1	0	0
Merrow_LC	61	**58**	0	1	2	0	**60**	1	0	**54**	0	0	0	1	0	1	0	0	0	1	0	2	0	2	0	0	0	0	0	2
Conan_ET	2	**2**	0	0	0	0	**2**	0	0	**2**	0	0	0	0	0	0	0	0	0	0	0	0	0	0	0	0	0	0	0	0
Chapaev3-1_AA	30	**27**	1	2	1	1	**24**	1	4	**25**	0	0	0	0	0	0	0	0	1	1	0	0	0	0	0	0	1	0	1	1
Chapaev3-2_AA	41	**40**	0	0	1	0	**37**	4	0	**36**	0	0	0	0	0	0	1	1	0	0	1	0	0	1	1	0	0	0	0	0
Chapaev3-3_AA	5	**4**	0	1	0	0	**5**	0	0	**4**	0	0	0	0	0	0	1	0	0	0	0	0	0	0	0	0	0	0	0	0
Chapaev3-1N1_AAe	137	**126**	1	8	2	0	**125**	1	11	**120**	2	0	0	0	1	0	2	0	0	0	0	2	0	3	1	0	1	2	1	2
Garfield _RP	3	**3**	0	0	0	0	**3**	0	0	**3**	0	0	0	0	0	0	0	0	0	0	0	0	0	0	0	0	0	0	0	0
MerrowN1_TO	2	**2**	0	0	0	0	**2**	0	0	**2**	0	0	0	0	0	0	0	0	0	0	0	0	0	0	0	0	0	0	0	0
Garfield _BM	6	**6**	0	0	0	0	**6**	0	0	**6**	0	0	0	0	0	0	0	0	0	0	0	0	0	0	0	0	0	0	0	0
Garfield N1_BM	58	**52**	1	3	2	0	**54**	3	1	**54**	0	0	0	0	1	0	0	0	0	0	0	0	0	1	0	0	0	2	0	0
Merrow_LF	3	**3**	0	0	0	0	**3**	0	0	**3**	0	0	0	0	0	0	0	0	0	0	0	0	0	0	0	0	0	0	0	0
Garfield _AR	2	**2**	0	0	0	0	**2**	0	0	**2**	0	0	0	0	0	0	0	0	0	0	0	0	0	0	0	0	0	0	0	0
Chapaev3-1_DA	33	**32**	0	0	1	0	**32**	0	1	**32**	0	0	0	0	0	0	0	0	0	0	0	0	0	1	0	0	0	0	0	0
Chapaev3-1_DW	18	**18**	0	0	0	0	**17**	1	0	**18**	0	0	0	0	0	0	0	0	0	0	0	0	0	0	0	0	0	0	0	0
Chapaev3-1_NVi	7	**7**	0	0	0	0	**7**	0	0	**7**	0	0	0	0	0	0	0	0	0	0	0	0	0	0	0	0	0	0	0	0
Chapaev3-2_NVi	2	**2**	0	0	0	0	**2**	0	0	**2**	0	0	0	0	0	0	0	0	0	0	0	0	0	0	0	0	0	0	0	0
Chapaev3-1N_FR	7	**6**	1	0	0	0	**7**	0	0	**7**	0	0	0	0	0	0	0	0	0	0	0	0	0	0	0	0	0	0	0	0
Chapaev3-1_SM	5	**5**	0	0	0	0	**5**	0	0	**4**	0	0	0	0	0	0	0	0	0	0	0	0	0	0	0	0	1	0	0	0
Chapaev3-1_HR	11	**10**	0	1	0	0	**11**	0	0	**9**	0	0	0	0	0	0	2	0	0	0	0	0	0	0	0	0	0	0	0	0
Chapaev3-2_HR	4	**4**	0	0	0	0	**3**	1	0	**4**	0	0	0	0	0	0	0	0	0	0	0	0	0	0	0	0	0	0	0	0
Chapaev3-3_HR	3	**3**	0	0	0	0	**3**	0	0	**2**	0	0	0	0	0	0	0	0	0	0	0	0	0	0	0	0	1	0	0	0
Chapaev3-N1_HR	9	**9**	0	0	0	0	**8**	1	0	**7**	0	0	0	0	0	0	0	0	0	0	1	0	0	0	0	0	0	0	0	0

Table S4 Pairwise comparison of *Merrow* nucleotide sequence identity in the studied species

	Merrow_LC	*Merrow_PM*	*Merrow_AT*	*Merrow_IF*	*Merrow_IP*	*Merrow_PS*	*MerrowN1_TO*
Merrow_PM	99.8						
Merrow_AT	98.1	98.2					
Merrow_IF	87.4	87.4	87.9				
Merrow_IP	82.8	82.6	ND	ND			
Merrow_PS	93.7	93.7	94.9	ND	ND		
MerrowN1_TO	94.4	94.4	92.0	ND	ND	91.0	
Merrow_LF	92.0	92.0	92.5	ND	79.3	90.8	88.7

Note: The species abbreviations for *Merrow* are as follows: PM, *Petromyzon marinus*; LC, *Lethenteron camtschaticum*; AT, *Acipenser transmontanus*; IF, *Ictalurus furcatus*; IP, *Ictalurus punctatus*; PS, *Polyodon spathula*; TO, *Thunnus orientalis*; LF, *Ladona fulva*. ND, not determined, as there was no or short (< 300 bp) overlap region shared by *Merrow* from two species.

Table S5 Pairwise comparison of *Garfield* nucleotide sequence identity in the studied species

	Garfield_RP	*Garfield_BM*	*Garfield_MB*	*Garfield_PX*	*Garfield_AR*	*Garfield_DP*
Garfield_BM	99.0					
Garfield_MB	98.2	97.9				
Garfield_PX	96.6	96.1	95.6			
Garfield_AR	98.1	97.7	97.5	96.4		
Garfield_DP	99.0	98.8	97.9	96.6	98.1	
GarfieldN1_BM	90.5	90.5	89.7	86.8	90.1	89.8

Note: The species abbreviations for *Garfield* are as follows: BM, *Bombyx mori*; RP, *Rhodnius prolixus*; MB, *Cotesia sesamiae Mombasa bracovirus*; PX, *Papilio xuthus*; AR, *Athalia rosae*; DP, *Danaus plexippus*.

Table S6 Pairwise comparison of *Conan* nucleotide sequence identity in the studied species

	ConanN1_AC	*Conan_ET*	*Conan_AC*
Conan_ET	97.6		
Conan_AC	88.7	87.6	
Conan N2_AC	98.3	97.6	87.8

Note: The species abbreviations for *Conan* are as follows: ET, *Echinops telfairi*; AC, *Anolis carolinensis*.

Table S7 Summary of non-synonymous (*dn*) and synonymous (*ds*) substitutions of *Merrow* and *Garfield* families.

**Transposons**	***dn***					***ds***					***dn/ds***				
Merrow	*Pm*	*Lc*	*Lf*	*At*		*Pm*	*Lc*	*Lf*	*At*		*Pm*	*Lc*	*Lf*	*At*	
P. marinus (Pm)															
L. camtschaticum (Lc)	**0.0000**					**0.0000**					**NA**				
L. fulva (Lf)	**0.0648**	**0.0648**				**0.1763**	**0.1763**				**0.3676**	**0.3676**			
Ac. transmontanus (At)	**0.0115**	**0.0115**	**0.0509**			**0.0088**	**0.0088**	**0.1934**			**1.3068**	**1.3068**	**0.2632**		
Garfield	*Ar*	*Dp*	*Px*	*Rp*	*Bm*	*Ar*	*Dp*	*Px*	*Rp*	*Bm*	*Ar*	*Dp*	*Px*	*Rp*	*Bm*
A. rosae (Ar)															
D. plexippus (Dp)	**0.0240**					**0.0437**					**0.5492**				
P. xuthus (Px)	**0.0446**	**0.0110**				**0.0718**	**0.0375**				**0.6212**	**0.2933**			
R. prolixus (Rp)	**0.0296**	**0.0083**	**0.0166**			**0.0379**	**0.0092**	**0.0248**			**0.7810**	**0.9022**	**0.6694**		
B. mori (Bm)	**0.0337**	**0.0105**	**0.0166**	**0.0090**		**0.0624**	**0.0183**	**0.0507**	**0.0154**		**0.5401**	**0.5676**	**0.3274**	**0.5844**	

Table S8 Summary of non-synonymous (*dn*) and synonymous (*ds*) substitutions of elongation factor 1-alpha (*EF-1α*) genes.

Gene	Amino acid divergence (%)	Nucleotide acid divergence (%)	*dn*	*ds*	*dn*/*ds*
EF-1α					
Bombyx versus *Rhodnius*	7.0	21.1	0.0553	3.4936	0.0158
Bombyx versus *Danaus*	2.4	13.2	0.0184	0.8388	0.0219
Bombyx versus *Papilio*	2.4	13.2	0.0151	0.8891	0.0170
Papilio versus *Danaus*	1.7	10.3	0.0115	0.5696	0.0202
Papilio versus *Rhodnius*	7.0	21.6	0.0000	NA*	NA*
Danaus versus *Rhodnius*	5.8	21.6	0.0000	NA*	NA*
Danaus versus *Athalia*	4.8	17.5	0.0425	1.2761	0.0333
Papilio versus *Athalia*	5.5	17.7	0.0501	1.2522	0.0400
Bombyx versus *Athalia*	5.5	18.7	0.0529	1.4106	0.0375
Rhodnius versus *Athalia*	5.7	21.4	0.0000	NA*	NA*

*SNAP does not give an output for *ds* when $p_s > 0.75$, suggesting substitution saturation.

Table S9 Summary of the effective number of codon (Nc) value of *Merrow*, *Garfield* and *Conan*

Transposon names	Nc
Merrow_PM	53.72
Merrow_LC	53.72
Merrow_LF	56.06
Merrow_AT	59.15
Garfield_RP	51.51
Garfield_BM	50.89
Garfield_DP	51.37
Garfield_PX	54.37
Garfield_AR	49.72
Conan_ET	49.58

Species abbreviations: PM, *Petromyzon marinus*; LC, *Lethenteron camtschaticum*; LF, *Ladona fulva*; AT, *Acipenser transmontanus*; RP, *Rhodnius prolixus*; BM, *Bombyx mori*; DP, *Danaus plexippus*; PX, *Papilio xuthus*; AR, *Athalia rosae*; ET, *Echinops telfairi*.
